# Supplementary material for: Rhodopsin molecular evolution from mouse to human phenylalanine 88 to leucine substitution enhances thermal stability and post-activation decay
Source: Sci Rep. 2026 Jan 26;16:3500. doi: 10.1038/s41598-025-32531-8 (PMC12847716; doi:10.1038/s41598-025-32531-8)
Supplement: Supplementary file 1 — Supplementary Information. [file 41598_2025_32531_MOESM1_ESM.pdf]

Supplementary Information

Rhodopsin molecular evolution from mouse to human Phenylalanine 88 to Leucine substitution enhances thermal stability and post-activation decay

Feifei Wang, Alexander V. Kolesnikov, Shinya Sato, Aneal Singh, Clint L. Makino, Pere Garriga, and Vladimir J. Kefalov

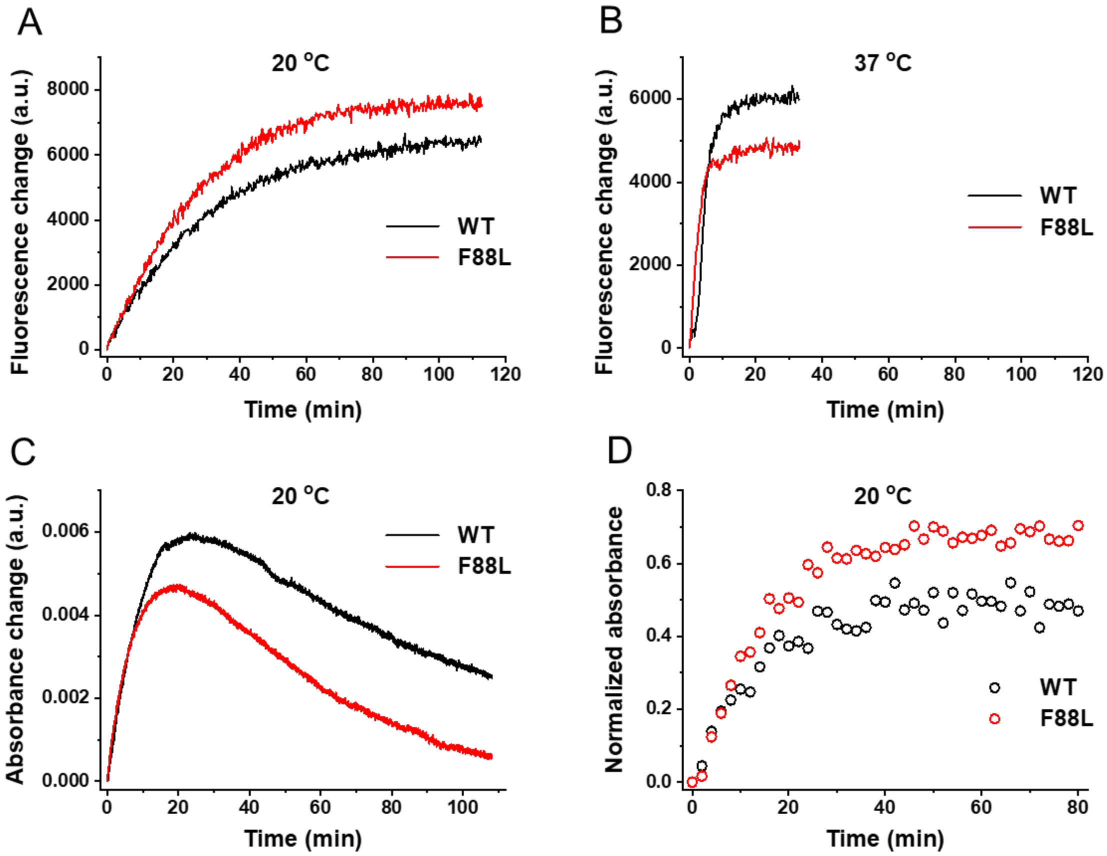

Supplementary Fig. 1. Representative original curves for the decay of Meta II at 20 ºC (**A**) or 37 ºC (**B**) obtained by fluorescence at 330 nm (for W265, see Methods) after near complete pigment bleach (summarized in Fig. 5A, B). (**C**) Representative raw traces for the kinetics of formation and decay of Meta III at 20 ºC, monitored by absorbance changes at 465 nm after near complete pigment bleach (summarized in Fig. 5C, D). (**D**) Representative data for regeneration of rhodopsin with exogenous 11-*cis*-retinal at 20 ºC measured by UV-visible spectrophotometry after near complete pigment bleach (summarized in Fig. 5E, F). The absorbance data were normalized for their respective pre-bleach rhodopsin levels.
